# Hematological and Biochemical Responses of Newly Formulated Primary Root Canal Obturating Material: An In Vivo Study

**DOI:** 10.7759/cureus.32685

**Published:** 2022-12-19

**Authors:** Prasanna T Dahake, Nilima Thosar, Alka Hande, Dipali A. Joshi, Amit Bhagat

**Affiliations:** 1 Department of Pediatric and Preventive Dentistry, Sharad Pawar Dental College and Hospital, Datta Meghe Institute of Medical Sciences (Deemed to be University) Sawangi, Wardha, IND; 2 Department of Oral Pathology and Microbiology, Sharad Pawar Dental College and Hospital, Datta Meghe Institute of Medical Sciences (Deemed to be University) Sawangi, Wardha, IND; 3 Central Research Lab, Datta Meghe Institute of Medical Sciences (Deemed to be University) Sawangi, Wardha, IND; 4 Dentistry, Mahatma Gandhi Institute of Medical Sciences, Wardha, IND

**Keywords:** zinc oxide, root canal filling materials, rats wistar, metapex, eugenol, dental pulp cavity

## Abstract

Background and objective

Any drug or medicinal agent, when implanted into the body, gets biotransformed by various organ systems and the toxic byproducts of this process alter the normal physiological process. In this experimental study, we aimed to quantify the safety of newly formulated primary root canal obturating material by investigating the hematological and biochemical parameters related to liver function.

Methodology

Forty-eight Wistar rats (weighing 250-350 grams) were classified into three groups (n=16) through random allocation. Preoperative blood samples were collected by puncturing the orbital venous plexus, the values of which were used as control. Zinc oxide eugenol (ZOE), calcium hydroxide iodoform paste (Metapex), and newly formulated triple antibiotic obturating paste (TAOP) were implanted (100 µg) into dorsal connective tissues. Blood samples on the seventh, 15th, and 30th postoperative days were evaluated respectively by analyzing hematological, hepatic, and, renal function tests for acute and chronic inflammatory responses.

Results

The intra-group and inter-group comparisons among all the test materials after seven days exhibited high significance in terms of hemoglobin (Hb), mean corpuscular volume (MCV), neutrophils, and serum glutamic-oxaloacetic transaminase (SGOT) (p<0.001), while others showed mixed responses (p<0.05 to p>0.05). After 15 days, the comparisons showed high significance with respect to packed cell volume (PCV), mean cell volume (MCV), and serum creatinine (p<0.001), while others showed significant to nonsignificant differences (p<0.05 to p>0.05). At the end of 30 days, all the parameters showed mixed responses (p<0.001 to p>0.05).

Conclusion

The newly formulated obturating material TAOP showed lower adverse hematological, hepatic, and renal effects in experimental animals compared to other test materials, with most parameters reverting to normal after 30 days.

## Introduction

Hematological parameters reflect the physiological functioning of the whole body and are hence imperative in the diagnosis and identification of any derailments in the structural and functional gradients of the human body coming into contact with chemicals [1]. Several hematologic indices, for instance, hemoglobin (Hb), red blood cells (RBC), hematocrit (Hct), and biochemical analyses are regularly utilized for accessing the oxygen-carrying capacity and another functional status of the blood, which ultimately can act as benchmark predictors for altered physiological functions [2]. Any drug or medicinal molecule when ingested or injected into the body is transferred by blood in the dissolved or undissolved form to various organ systems. Depending on the molecular structure of the element, it is digested and biotransformed in various systems and tissues. The liver and kidneys [3] are the primary organs where most of the absorbed elements are processed, absorbed, and prepared for excretion. Some of the chemical, biological or biochemical agents have the potential to alter the normal physiological process of the body due to their molecular alteration during biotransformation. While it may benefit multiple organ systems, it can also harm them if not monitored closely and adequately. Such alterations are usually recorded as altered values of organ function tests [4,5]. Several reports have indicated the potential of some root canal-filling materials to alter the cell physiology they come into contact with [6]. Such materials impose local, systemic as well as transport system toxicity too, after absorption and biotransformation in various organs of the human body [7].

Under the given circumstances, either the overload of or high exposure to chemical agents from such materials can disturb the cellular response by damaging cell membranes, and mitochondria to intracellular organelles, and cause the breakage of DNA strands [8,9]. An experimental study [10] conducted on zinc oxide eugenol (ZOE), and calcium hydroxide iodoform paste has documented that these materials impose toxicity. Authors have recently developed a novel gel formulation containing clindamycin, metronidazole, and doxycycline added at a specific concentration with chitosan as well as Carbopol as a vehicle to be used as an obturating material in primary teeth root canals. Adverse effects of the previously mentioned obturating materials on various cells in close vicinity to radicular and periradicular regions have been investigated extensively [11,12]. However, the hematological parameters of these materials have rarely been studied. Hence, We believe it is a prerequisite to evaluate the effects of conventional as well as newly formulated obturating materials on hematological parameters in experimental animals for a better understanding of the pharmacokinetics and pharmacodynamics of these materials before proceeding to human trials. The current experimental study aimed to explore the effects of three different primary root canal obturating materials on hematological and a few biochemical parameters in experimental animals, i.e., Wistar rats.

## Materials and methods

Study design

The present experimental animal study was conducted at the Department of Pharmacology, Department of Pediatric and Preventive Dentistry, and Institutional animal house after gaining approval from Institutional Ethical Committee and Institutional Animal Ethical Committee. The study's ethical approval protocol was approved by the Institutional Animal Ethical Committee Board Ref No. DMIMSDU/IAEC/2015-16/1, based on the CPCSEA (Committee for the Purpose of Control and Supervision of Experiments on Animals) guidelines.

Procurement and sample size calculation of animals for study

In this experimental study, 48 colony-bred adult male and female Wistar rats (24 each) with weights ranging from 250-350 grams were classified equally into three groups (n=16) through random allocation. Animals required for the study were procured from the institutional animal house and kept in the laboratory for two weeks, to make them accustomed to the environment before starting the experiment. The animals were kept in specially designed polypropylene cages under a well-structured, adequately ventilated, light/dark (12 hr:12 hr) cycle at an average temperature of 22 ±1 °C. Animals were fed with rat food and given sufficient water as specified in the CPCSEA guidelines [13] ad libitum before and during the experiment.

Collection of preoperative blood samples

Sedation of the animals was achieved by inducing an appropriate dose of ketamine and xylazine based on the mg/kg formula [14,15]. It was decided to use the left and right eye alternatively for preoperative (00 days) as well as postoperative (seventh, 15th, and 30th days) blood collection to minimize the ophthalmic damage and reduce stress induction. After securing adequate anesthesia, the left eye was applied with the topical ophthalmic anesthetic gel before inducing the bleeding. The animal was scruffed with a forefinger and thumb of the left hand to pull the periophthalmic skin tightly. A sterile glass capillary was inserted into the medial canthus of the eye (at an angulation of 30 degrees to nasal aperture), with slight digital pressure to enter the orbital venous plexus [13,16]. As the plexus was punctured, blood running down through the capillary tube was collected in a bulb containing anticoagulant ethylenediaminetetraacetic acid (EDTA) for hematological analysis and a plain bulb containing clot activator for biochemical studies, respectively [17]. The tubes were shaken gently to mix the contents of the bulbs properly. When the required blood quantity was gathered from the plexus, the tube was removed, which was precisely followed by wiping the eye with saline-soaked sterile cotton. Further bleeding from the eye was arrested by applying gentle digital pressure with sterile wet cotton [13]. The animals were observed for approximately 30 minutes to evaluate for any postoperative or periorbital lesions.

Meanwhile, to avoid desiccation and dissociation, the blood samples were sent immediately to the central research laboratory for further analysis. Hematological analyses were done using an automatic hematological analyzer (ABX Micros ESV 60, Horiba Medical, Kyoto, Japan). Hematological parameters included in this study were as follows: Hb, packed cell volume (PCV), RBC count, mean corpuscular volume (MCV), mean corpuscular hemoglobin (MCH), mean corpuscular hemoglobin concentration (MCHC), platelets count, total leucocyte count (TLC) and differential leukocyte count (DLC). The DLC was done to evaluate neutrophil, lymphocyte, monocyte, and eosinophil count after staining the blood smears with H&E (hematoxylin and eosin) staining, counting 100 cells per view [17,18]. For biochemical analysis, samples were centrifuged with a research centrifuge machine (Remi Scientific Pvt. Ltd., Mumbai, India) at 1500 × g for 10 minutes to obtain serum and stored at −20 °C [17]. The biochemical tests performed using an automated biochemistry analyzer (Prietest Touch, Robonik Pvt. Ltd, Thane, India) were as follows: serum urea (Autospan Liquid gold Urea test kit, Arkray Healthcare Pvt Ltd, Surat, India), serum creatinine (Autospan Liquid gold Creatinine test kit, Arkray Healthcare), serum glutamic-oxaloacetic transaminase (SGOT) (Truechemie SGOT test kit, Athenese-Dx Pvt Ltd, Chennai, India) and serum glutamic-pyruvic transaminase (SGPT) (Truechemie SGPT test kit, Athenese-Dx Pvt Ltd, Chennai, India). The values from preoperative blood samples were analyzed and used as controls. All the animals were given appropriate analgesics to alleviate pain, followed by adequate rest, with ad libitum food and water to enhance healing and stress relief for two weeks [13].

The test materials used in this study are listed in Table 1.

**Table 1:** Test materials used in the study

Sr. no.	Animal group	Test material
1.	Group A	Zinc oxide eugenol (ZOE)
2.	Group B	Calcium hydroxide Iodoform paste (Metapex)
3.	Group C	Triple antibiotic obturating paste (TAOP)

Preparation of experimental materials for implantation in rat tissues

Zinc oxide (ZO) powder and eugenol (E) were dispensed and mixed on a clean glass slab according to the manufacturer’s instructions, and at the ratio mentioned by Tchaou et al. [19] to achieve creamy consistency [20]. ZOE was inserted using a paste carrier, while Metapex and triple antibiotic obturating paste (TAOP) were inserted using high-pressure syringes respectively, into sterile polyethylene terephthalate (PET) tubes (10 mm length x 1 mm internal diameter) at a quantity of approximately 100 µg [14,15].

Method for the experimental procedure

The material-loaded PET tubes were implanted into rats' back connective tissues after achieving adequate sedation. Animals were monitored carefully for uneventful recovery from anesthesia and during the postoperative phase [14,15]. All the animals were taken care of as per the guidelines of CPCSEA.

Collection of postoperative blood samples

To analyze the post-operative changes, blood samples were collected after securing adequate anesthesia. All the procedures for blood collection were followed as mentioned previously and blood samples were collected on the seventh (right eye), 15th (left eye), and 30th (right eye) postoperative day for evaluating acute and chronic inflammatory responses through hematological parameters, liver as well as kidney function test respectively.

Statistical analysis

All the data for each sample and test was entered in a Microsoft Excel sheet followed by statistical analysis using IBM SPSS Statistics for Windows, version 22.0 (IBM Corp., Armonk, NY) using descriptive statistics. The analyzed data were presented as mean ±standard deviation (SD). For intra-group comparison, paired t-test was employed, while for inter-group comparison, one-way ANOVA followed by Tukey's post-hoc Honest Significant Difference (HSD) test was used. The confidence level was set at 95% (p<0.05).

## Results

Hematological parameters

The hematological parameters (mean blood parameters) were analyzed at the end of 00, seven, 15, and 30 days (Table 2). It was observed that at the end of seven days, animals implanted with ZOE and Metapex showed decreased mean Hb concentration, compared to TAOP against the normal range, which did not enhance till the 15th postoperative day. However, after 30 days, a slight enhancement was observed in the ZOE and Metapex groups. The mean PCV decreased initially in both ZOE and Metapex groups at the end of seven and 15 days, but it remained relatively unaffected in TAOP group experimental animals, and later it increased gradually at the end of 30 days. The mean RBC count also decreased initially in ZOE and Metapex groups compared to the TAOP group after seven and 15 days, which gradually increased to near-normal levels at the end of the 30th postop day. The mean MCV also decreased significantly in ZOE and Metapex group animals as compared to TAOP group animals till the 15th postoperative day and gradually increased by the 30th day to normal levels. The mean MCH was not altered in any of the experimental group animals at any period and remained almost unchanged. The mean MCHC was also found unchanged due to any of the material inserted in the animals' bodies at the end of seven and 15 days. But, surprisingly, it was found slightly increased in Metapex and TAOP group animals at the end of 30 days. The mean platelet count was initially found to have increased for ZOE and Metapex group animals as compared to TAOP group animals at the end of seven days, which suddenly fell in the ZOE group at the end of 15 days and in the Metapex group at the end of 30 days.

**Table 2:** Blood parameters of experimental animals at 00, 07, 15, and 30 days SD: standard deviation; ZOE: zinc oxide eugenol; TAOP: triple antibiotic obturating paste; Hb: hemoglobin; PCV: packed cell volume; RBC: red blood cell count; MCV: mean corpuscular volume; MCH: mean corpuscular hemoglobin; MCHC: mean corpuscular hemoglobin concentration

Parameters	Control group preoperative, mean ±SD	Postoperative 7 days, mean ±SD			Postoperative 15 days, mean ±SD			Postoperative 30 days, mean ±SD		
		ZOE	Metapex	TAOP	ZOE	Metapex	TAOP	ZOE	Metapex	TAOP
Hb (g/dL)	16.06 ±0.82	12.03 ±0.54	13.08 ±0.78	14.54 ±0.50	11.91 ±0.45	13.34 ±0.91	14.22 ±0.75	12.64 ±0.67	13.49 ±0.77	14.23 ±0.82
PCV (%)	45.50 ±1.43	36.98 ±0.58	37.97 ±0.76	42.64 ±1.94	37.29 ±0.72	39.90 ±1.67	41.61 ±3.60	38.19 ±1.68	39.73 ±2.22	42.09 ±2.43
RBC (x10^6^/µl)	8.71 ±0.59	7.04 ±0.41	7.41 ±0.46	8.41 ±0.61	7.62 ±0.47	8.49 ±0.82	9.09 ±0.57	7.89 ±0.54	8.53 ±0.76	9.01 ±0.66
MCV (fL)	75.16 ±9.58	62.11 ±2.90	67.02 ±3.83	74.43 ±3.07	62.43 ±2.23	69.08 ±4.42	73.68 ±4.06	65.65 ±3.64	69.96 ±4.05	72.91 ±4.13
MCH (pg)	18.94 ±1.24	20.38 ±1.94	20.36 ±1.64	20.40 ±1.32	19.48 ±1.37	19.80 ±1.28	20.19 ±1.37	19.84 ±1.36	19.70 ±1.17	21.85 ±0.84
MCHC (g/dL)	29.57 ±0.97	29.75 ±1.31	29.82 ±1.27	29.15 ±1.78	29.17 ±1.02	29.70 ±0.80	29.21 ±1.12	29.13 ±1.18	30.60 ±0-.93	31.28 ±1.05
Platelet (x10^5^/µl)	7.84 ±0.79	9.66 ±0.97	8.64 ±2.40	7.44 ±0.79	6.92 ±0.34	7.26 ±0.39	7.49 ±0.91	7.09 ±0.58	6.67 ±0.41	7.63 ±0.81

Differential leucocyte count parameters

Table 3 shows the differential leucocytic counts in all the experimental animals for observational durations. The mean TLC was found significantly increased in animals in the ZOE group, followed by those in the Metapex group while slightly decreased in TAOP group animals. However, it suddenly decreased in all the animals after 15 days and came to a near-normal range by 30 days. The mean neutrophil count highly increased in both ZOE and Metapex group animals compared to the TAOP group, which gradually decreased over time after 15 days and came to near-normal by 30 postoperative days. The mean lymphocyte count was initially unaltered at the end of seven days but gradually increased in animals of all the groups after 15 days and significantly after 30 days. The mean monocyte count was initially unchanged after seven days but gradually increased two-fold and almost by four times at the end of 30 days. The mean eosinophil count also showed a similar pattern of alteration on the 15th and 30th days of postoperative observation.

**Table 3:** Differential leucocyte counts in experimental animals at 00, 07, 15, and 30 days SD: standard deviation; ZOE: zinc oxide eugenol; TAOP: triple antibiotic obturating paste; TLC: total leucocyte count

Parameters	Control group preoperative, mean ±SD	Postoperative 7 days, mean ±SD			Postoperative 15 days, mean ±SD			Postoperative 30 days, mean ±SD		
		ZOE	Metapex	TAOP	ZOE	Metapex	TAOP	ZOE	Metapex	TAOP
TLC (x10^3^/µl)	8.38 ±0.63	16.49 ±7.17	11.17 ±1.39	7.24 ±0.80	7.56 ±0.88	7.71 ±0.60	7.51 ±0.67	8.42 ±0.50	7.77 ±0.52	7.72 ±0.79
Neutrophils (%)	8.82 ±1.14	27.70 ±1.71	21.80 ±1.74	19.06 ±0.56	16.93 ±0.80	16.34 ±0.71	15.60 ±0.81	8.99 ±0.57	7.72 ±0.45	7.88 ±0.71
Lymphocytes (%)	63.27 ±0.83	63.31 ±0.87	63.12 ±2.12	63.94 ±1.33	70.72 ±1.77	70.56 ±1.44	69.22 ±1.38	79.36 ±2.70	79.27 ±2.04	79.52 ±1.62
Monocytes (%)	1.10 ±0.42	1.04 ±0.29	1.07 ±0.56	0.96 ±0.46	2.18 ±0.50	2.01 ±0.52	1.59 ±0.92	4.50 ±0.82	3.86 ±0.68	3.89 ±0.33
Eosinophils (%)	1.01 ±0.35	1.27 ±0.46	0.96 ±0.44	1.04 ±0.52	2.92 ±0.45	2.64 ±0.31	2.95 ±0.26	5.16 ±0.50	4.66 ±0.57	4.73 ±0.54

Biochemical parameters

Table 4 shows the biochemical parameters in all the experimental animals evaluated for test materials during observational durations. The mean serum urea in ZOE and Metapex groups was found to have increased significantly as compared to TAOP group animals after seven days. It gradually declined over time, by the end of 30 days. The mean serum creatinine was also found to have increased significantly after seven days but decreased slightly after 15 days. However, it was found to have significantly decreased at the end of 30 days (to half after the 30th postoperative day). When the mean SGOT was evaluated, it was found to be increased significantly at the end of seven days but gradually declined by the 15th postoperative day, and reduced to less than normal for all the groups on the 30th day. The mean SGPT values were significantly increased in ZOE group animals but were relatively less in the Metapex group and were found to be the least in TAOP group animals. It gradually decreased in all three groups but was found at a rate of almost double the normal values after 30 days.

**Table 4:** Biochemical parameters in experimental animals at 00, 07, 15, and 30 days SD: standard deviation; ZOE: zinc oxide eugenol; TAOP: triple antibiotic obturating paste; S. urea: serum urea; S. Crt: serum creatinine; SGOT: serum glutamic-oxaloacetic transaminase; SGPT: serum glutamic-pyruvic transaminase

Parameters	Control group preoperative, mean ±SD	Postoperative 7 days, mean ±SD			Postoperative 15 days, mean ±SD			Postoperative 30 days, mean ±SD		
		ZOE	Metapex	TAOP	ZOE	Metapex	TAOP	ZOE	Metapex	TAOP
S. urea (mg/dL)	18.17 ±1.92	30.98 ±5.91	30.25 ±4.06	25.51 ±1.57	27.76 ±1.06	24.27 ±1.37	24.25 ±0.88	24.44 ±0.75	22.62 ±5.53	22.92 ±1.29
S. Crt (mg/dL)	0.48 ±0.14	0.89 ±0.11	0.75 ±0.06	0.77 ±0.11	0.70 ±0.11	0.59 ±0.08	0.56 ±0.10	0.29 ±0.06	0.24 ±0.08	0.22 ±0.14
SGOT (IU/ml)	110.07 ±22.63	169.81 ±12.89	154.33 ±14.33	128.09 ±14.81	108.05 ±8.82	107.60 ±9.34	107.10 ±8.52	89.58 ±5.71	88.25 ±6.22	88.50 ±7.37
SGPT (IU/ml)	36.67 ±7.49	101.59 ±14.63	85.58 ±13.96	58.73 ±7.31	66.24 ±4.22	64.71 ±3.87	65.09 ±2.48	66.01 ±6.38	68.08 ±4.25	68.05 ±5.11

Table 5 presents inter-group comparisons of the hematological, leucocyte count, and biochemical parameters, which, as shown in Tables 2-4, showed a statistically highly significant difference for Hb, MCV, neutrophil, and SGOT at the end of seven days in all groups. While other parameters showed either significant or non-significant statistical differences in terms of inter-group comparisons, the inter-group comparisons at the end of 15 days showed a statistically highly significant difference for PCV and MCV. However, at the end of 30 days, almost all the parameters showed a statistically non-significant difference.

**Table 5:** Inter-group and intra-group comparisons of blood parameters in experimental animals at 00, 07, 15, and 30 days *Significant. **Highly significant. ^#^Non-significant F-test: ANOVA (inter-group comparisons). T-test: intra-group comparisons ZOE: zinc oxide eugenol; TAOP: triple antibiotic obturating paste; Hb: hemoglobin; PCV: packed cell volume; RBC: red blood cell count; MCV: mean corpuscular volume; MCH: mean corpuscular hemoglobin; MCHC: mean corpuscular hemoglobin concentration; TLC: total leucocyte count; S. urea: serum urea; S. Crt: serum creatinine; SGOT: serum glutamic-oxaloacetic transaminase; SGPT: serum glutamic-pyruvic transaminase

Parameter	F-test	T-test - 07 days (p-values)			F-test	T-test - 15 days (p-values)			F test	T-test - 30 days (p-values)		
		ZOE vs. Metapex	Metapex vs. TAOP	ZOE vs. TAOP		ZOE vs. Metapex	Metapex vs. TAOP	ZOE vs. TAOP		ZOE vs. Metapex	Metapex vs. TAOP	ZOE vs. TAOP
Hb	66.34	0.001**	0.0001**	0.0001**	40.98	0.0004**	0.01^#^	0.00001**	17.65	0.002*	0.01^#^	0.00001**
PCV	94.15	0.00**	5.04^#^	3.09^#^	13.97	0.0003**	0.000001**	0.00001**	13.55	0.04^#^	0.01*	0.00001**
RBC	31.86	0.02^#^	0.0001**	0.000003**	21.63	0.001**	0.02*	0.00001**	11.44	0.01*	0.07^#^	0.0001**
MCV	56.85	0.0003**	0.00001**	0.0000001**	37.45	0.0001**	0.005**	0.0001**	13.7	0.004*	0.05*	0.0001**
MCH	1.69	0.14^#^	0.88^#^	0.08^#^	1.14	0.5^#^	0.41^#^	0.15^#^	17.11	0.76^#^	0.0001**	0.00002**
MCHC	0.99	0.88^#^	0.23^#^	0.29^#^	1.39	0.11^#^	0.17^#^	0.91^#^	17.15	0.001**	0.06^#^	0.00001**
Platelet	8.1	0.13^#^	0.07^#^	0.00001**	3.59	0.01*	0.37^#^	0.03*	9.44	0.02*	0.0002**	0.04*
TLC	19.18	0.01^#^	0.0001**	0.00002**	0.31	0.60^#^	0.39^#^	0.85^#^	6.4	0.001**	0.84^#^	0.005**
Neutrophils	149.48	0.00001**	0.0001**	0.00001**	11.84	0.04*	0.01*	0.0001**	22.04	0.00001**	0.47^#^	0.00003**
Lymphocytes	1.27	0.74^#^	0.20^#^	0.12^#^	4.6	0.78^#^	0.01*	0.01*	0.06	0.91^#^	0.70^#^	0.84^#^
Monocytes	0.28	0.84^#^	0.53^#^	0.55^#^	3.2	0.37^#^	0.12^#^	0.03*	5.08	0.02*	0.89^#^	0.01*
Eosinophils	1.9	0.06^#^	0.63^#^	0.19^#^	3.85	0.05*	0.004**	0.79^#^	4.1	0.01*	0.71^#^	0.03*
S. urea	7.9	0.69^#^	0.0001**	0.001**	51.83	5.86^#^	9.25^#^	2.88^#^	1.4	0.20^#^	0.83^#^	0.0003**
S. Crt	10.73	0.0005**	0.36^#^	0.0005**	9.85	0.002**	0.41^#^	0.0004**	2.14	0.04*	0.7^#^	0.08^#^
SGOT	36.15	0.003**	0.0002**	0.00002**	0.04	0.89^#^	0.87^#^	0.76^#^	0.19	0.53^#^	0.91^#^	0.65^#^
SGPT	13.93	0.004**	0.10^#^	0.00001**	0.78	0.29^#^	0.74^#^	0.35^#^	0.8	0.29^#^	0.99^#^	0.32^#^

## Discussion

The invention of new materials needs exploration through specific preclinical protocols to determine their safety index before initiating clinical trials. In the experimental and therapeutic domains, such materials are expected to exhibit optimum antimicrobial and minimal cytotoxic effects to be deemed acceptable [21]. Although bioresorbable, the inadvertent plunging of such materials into the periradicular areas leads to inflammatory and immunological responses [22,23]. Hence, the material with optimally suitable properties is preferred by clinicians. The obturating material (TAOP) invented by the authors in the present study has been investigated previously for in vitro cytotoxicity profile [24]. This newly invented material (TAOP) contains three antibiotics, viz., clindamycin, doxycycline, and metronidazole, dispersed in the chitosan-poloxamer-Carbopol base [25] at a concentration of 5:5:1 w/w, based on the results obtained from previous studies [25,26].

For a better understanding of the newly designed therapeutic drugs, animal models have been used for several decades to evaluate toxicity parameters and determine therapeutic doses [27]. In the present study, Wister rats were used to evaluate in vivo hematological and biochemical responses of the implanted test materials for a better understanding of their pharmacokinetics and pharmacodynamics. Altered hematological and biochemical parameters are considered to reflect the dysfunction of vital organs and the hemodynamic mechanism of the body, concerning for adverse effects or toxicities induced by the toxic compounds released during the biotransformation of test materials in the animal and human body [28]. The hematological system is one of the most sensitive systems reflecting the physiological and pathological status of body function against test agents [29]. Clinical biochemical analyses are conducted to investigate the possible influence of all the test agents on the hepatic and renal functions of test animals. The liver is the primary organ for drug metabolism and excretion, and the evaluation of certain markers like SGOT and SGPT can help determine the hepatic safety profile of the test agent, which mimics human hepatotoxicity directly [27]. The renal system is highly susceptible to various toxins and toxic byproducts being filtered through glomerular tubules secondary to a large amount of blood flowing through it and accumulating there. The levels of serum creatinine and urea are classically used for diagnosing renal system damage [30].

In the present experimental study, three different types of primary root canal obturating material, from different compositional backgrounds, have been evaluated. The first and second are inorganic in origin, viz., the conventional and most commonly used ZOE, and calcium hydroxide iodoform (Metapex) [31,32]. The third test material utilized here was the organic excipients matrix incorporated multi-antimicrobial [26,33] novel polymeric gel [24]. As all of them are from different compositional backgrounds, the physiological responses exerted by the host tissues are bound to be variable. The responses in the form of intense chronic inflammatory reactions cause numerous altered cellular functions like neutrophil dysfunction, proliferation adhesion as well as the viability of cells secondary to inflammatory cytokines release [34]. On the other hand, due to poor phagocytosis, and persistent residual remnants, they either hamper the eruptive path of permanent teeth [35] or get degraded readily by tissue exposure rendering empty root canals susceptible to reinfection [36].

The ZOE used for obturation primarily contains zinc oxide (inorganic powder) admixed with eugenol (a phenolic extract), from certain essential oils, like clove, nutmeg, cinnamon, and bay leaf. Being similar to safroles and estragole chemically, the proven carcinogens, eugenol can also impose biological and biochemical mechanisms of mutagenesis or carcinogenesis. The different action mechanisms through which eugenol can induce toxicity are as follows: altered ionic homeostasis; specific lesions of the cell plasma membrane; and oxidative stress (OS) generation. The eugenol also depletes intracellular sulfhydryl (SH) tripeptide and glutathione (GSH) warranting antioxidant cellular protection. Former in vitro experiments have also described it as a cytotoxic agent to various cells line like mouse fibroblast - L929, rat hepatic cells, human dental pulp cells [37] as well as oral mucosal fibroblasts [38]. Similar effects have been observed in rat liver and lung [39]. The reason for the altered platelet profile here can be attributed to antiplatelet activity exhibited by eugenol, through thromboxane A2-dependent cyclooxygenase (COX) inhibition [40]. The altered hepatic function might be due to eugenol diminishing intracellular glutathione transferase through reduced cytosolic Ca++ [41], or the xenobiotic molecule bioactivan and its carcinogenic metabolite via cytochrome P450 enzymes such as CYP1A2, CYP2A6, and CYP2C9 and sulfotransferases [42] subsequently leading to hepatocellular injury. The altered renal functions in ZOE group animals are ascribed to eugenol’s inhibitory effects on intracellular Na+/K+ ATPase activity, diminishing ATP level and mitochondrial function [43] and thereby causing morphological alteration and renal cell apoptosis [44]. The changes observed in the blood parameters might be due to a combination of the above mechanisms, causing increased levels of aspartate aminotransferase, alanine aminotransferase, and total bilirubin levels irrespective of the concentration [45]. The other cytotoxic responses shown by eugenol at high doses might be attributed to its genotoxic effects without DNA damage, but more of fixation-like effects by cell machinery damage particularly. It also affects the expression of genes that are involved in inflammation, apoptosis followed by chronic degenerative diseases, and ultimately malignant transformation [46].

The second test material Metapex (calcium hydroxide iodoform paste), after degradation in the exposed tissues, releases iodine, which is lipophilic and cross-cell membranes, particularly the blood-brain barrier, and is implicated in a wide range of neurological problems. Rather, it shows pronounced generalized toxicity due to the production of carbon monoxide (CO) through the iodine analog of phosgene, OCI2, metabolism [7,47]. The hematological and biochemical changes observed in Metapex group animals can be attributed to reasons cited by Sell and Reynolds [8]. The iodoform present in this test material affects hepatic cells in the form of decreased oxidative demethylation of microsomes, and degranulation of the granular endoplasmic reticulum. It induces pleomorphism and alteration of mitochondria, dilatation of the Golgi apparatus, and conglomeration of the cytoplasmic membrane, ultimately causing centrilobular necrosis. The effects seen on hematological and biochemical parameters of the experimental animals can be attributed to the iodoform byproduct liberated after its biotransformation as cytotoxic and genotoxicity effects due to single- or double-strand breakage of animal cell DNA indicating its mutagenic and carcinogenic character [48].

The third test material TAOP contains antibacterial agents that are otherwise used effectively for antimicrobial therapy of periodontal disease. In addition to their antimicrobial effects, they can modulate host immune system cells directly [49]. Clindamycin plays a positive role in the immunomodulation effect by enhancing microbial chemotaxis oxidative burst of neutrophils, as well as a positive effect on phagocytosis. [49,50]. Doxycycline, apart from its antimicrobial effect, exerts pleiotropic functions. Doxycycline demonstrates anti-inflammatory as well as immunomodulatory properties, specifically WBC proliferation and function, cytokine synthesis, and matrix metalloproteinase (MMP) activity [51,52]. It also enhances neutrophil chemotaxis and its phagocytic metabolic functions. Additionally, it inhibits lymphocytic proliferation, Fas/Fas ligand-mediated apoptosis induction in B and T cells particularly [53,54], along with decreased immunoglobulin secretion by activated B lymphocytes [55]. Metronidazole, apart from its antimicrobial effect, helps to diminish inflammatory progressions by activating a local immune reaction. It halts pavementing and migration of leukocytes from the central bloodstream to endothelium and prevents cell adhesion. It inverts leukocytic attachment and emigration responses by reducing rolling velocity [56]. When it contacts the monocytes, it decreases microbial endotoxin stimulated α-TNF, and thus the immunosuppression of the macrophage function [57]. The filers/excipients such as chitosan and Carbopol used in the formulation of the TAOP are organic compounds that have been employed in numerous drug delivery systems in the field of pharmaceutics and modern medicine. These agents have already been found to exert high biocompatibility and minimal toxicity during their usage as a part of the drug delivery system [24]. These agents yield thermo-modulated in-situ hydrogel (TSHG), which has been demonstrated to enhance fibroblast growth, macrophage activation, and hemostasis, as well as excellent wound-sealing properties with extended drug release [25].

Looking at the results obtained from the experiment, it can be stated that TAOP has shown relatively low-intensity responses when compared to ZOE and Metapex. The reason can be attributed to the experimental finding mentioned in the previous study [58]. The hematological alterations seen with the TAOP group animals were relatively minimal than the control group, with a slight decrease in Hb, PCV, MCV, and platelet count contrary to unaltered MCH and MCHC values. The neutrophils increased initially but came down to normal. The lymphocyte count was near-normal initially, but gradually increased showing lymphocytosis. The monocytes also showed an upsurge in all three experimental groups, with more accentuated results in the ZOE group, which might be attributed to the high response of the cell to eugenol content in it [40]. The alteration in the eosinophils was noted gradually with the highest in the ZOE group and lowest in the Metapex group, which might be attributed to the high reactive composition of the test materials in the other two groups than Metapex. The decline in the value of the hematological parameter can be a result of disturbed hematopoietic system activity as it is highly sensitive to toxic byproducts created in the animal body and reflects in the form of decreased release of RBCs in circulation, and hence accountable for a radical decline in RBC count.

In this study, the hepatic parameters such as SGOT and SGPT also showed a significant upsurge from their normal values at the end of the first week, which gradually decreased but did not become normal at the end of 30 days. The parameters associated with the renal functions, i.e., serum urea and serum creatinine showed a sharp uprise during the first week, but gradually decreased at the end of 30 days. The reason for this can be explained by the high degree of stress generated on the animal body by the test agents, particularly ZOE, due to high inflammation as well as their biotransformation and excretion, causing damage to the hepatic cells as mentioned by Escobar-García et al. [40]. Several studies indicate that the accumulation of toxic byproducts in the hepatic and renal system has a high capacity to induce damage to the respective systems leading to altered expression of the organ system biomarkers seen in the results.

In summary, TAOP implantation at the given doses did not induce any major variations in the hematological, hepatic as well as the renal profile of the experimental animals as compared to ZOE and Metapex. The changes observed were also not significantly toxic, and hence the combination TAOP at the given dose can be considered safe for tested systemic profiles of the experimental animals. The favorable results of the present in vitro study in terms of hematological and biochemical evaluations can be advantageous for conducting human trials in the future.

Limitations of the study

Though this study is an in vivo toxicological evaluation study mimicking the human body, it has a few limitations; primarily, the animal models used here are not congruent with the human body system completely for all given clinical conditions. Only hepatic and renal systems were explored in this study, due to time and financial constraints. Finally, this was a short-term study and hence the findings are not representative of long-term systemic toxicological data.

## Conclusions

Based on our findings, the implantation of the newly formulated obturating material TAOP into the animal body has relatively minimal adverse effects on hematological, hepatic, and renal parameters compared to other test materials. The majority of the parametric alterations reverted to normal after 30 days of test material insertion. We recommend further long-term in vivo and human clinical trials for a better understanding of the material's action mechanism as well as toxicity profile with regard to hematological, hepatic, renal as well as other systems.
